# First discovery of Holocene cryptotephra in Amazonia

**DOI:** 10.1038/srep15579

**Published:** 2015-10-23

**Authors:** Elizabeth J. Watson, Graeme T. Swindles, Ivan P. Savov, Karen L. Bacon

**Affiliations:** 1 School of Geography, University of Leeds, Leeds, LS2 9JT, UK; 2 School of Earth and Environment, University of Leeds, Leeds, LS2 9JT, UK

## Abstract

The use of volcanic ash layers for dating and correlation (tephrochronology) is widely applied in the study of past environmental changes. We describe the first cryptotephra (non-visible volcanic ash horizon) to be identified in the Amazon basin, which is tentatively attributed to a source in the Ecuadorian Eastern Cordillera (0–1°S, 78-79°W), some 500-600 km away from our field site in the Peruvian Amazon. Our discovery 1) indicates that the Amazon basin has been subject to volcanic ash fallout during the recent past; 2) highlights the opportunities for using cryptotephras to date palaeoenvironmental records in the Amazon basin and 3) indicates that cryptotephra layers are preserved in a dynamic Amazonian peatland, suggesting that similar layers are likely to be present in other peat sequences that are important for palaeoenvironmental reconstruction. The discovery of cryptotephra in an Amazonian peatland provides a baseline for further investigation of Amazonian tephrochronology and the potential impacts of volcanism on vegetation.

Tephrochronology (dating sedimentary sequences using volcanic ash layers) is a particularly useful method for dating and correlating records of past environmental change^1^,^2^,^3^. Although the majority of volcanic ash (tephra) falls out close to the volcanic source, fine ash (<1 mm) can have an atmospheric residence time in the region of hours to months, during which tephra may be transported thousands of kilometres^4^. In high concentrations fine ash is a hazard for the health of humans and animals^5^ and even far from the volcanic source ash can be present in concentrations which can induce engine failure in modern jet aircraft^6^.

Following the initial discovery of microscopic tephra shards from Icelandic volcanoes in distal lakes and peatlands of Ireland and Scotland^7^,^8^, such invisible isochrons, commonly referred to as ‘cryptotephras’ have been identified in ice cores, terrestrial and marine sediments^9^,^10^,^11^. Cryptotephras can often be linked to a source region or even specific eruption(s) based on their glass geochemistry. Advances in geochemical analysis techniques, predominantly through Electron Probe Micro Analysis (EPMA) now allow for precise and accurate analysis with beam sizes as small as 3 μm^12^. Cryptotephra layers in distal archives are predominantly used as correlation and dating tools; however they can also provide insights into past volcanic activity otherwise buried by younger deposits or eroded in the proximal (near vent) area. Tephra layers which transgress continental boundaries^13^,^14^ provide the opportunity for the correlation of palaeoenvironmental records over large distances. Cryptotephra studies have focussed predominantly on northern latitudes of Europe, although cryptotephras have also been identified in many other regions for example China^15^, North America^16^, New Zealand^17^ and Far East Russia^18^. There have been several studies of macroscopic tephra layers in South America e.g.^19^,^20^, but cryptotephra studies have been confined to the regions of Argentina and Patagonia^21^,^22^. To the authors’ knowledge there have been no previous published studies of cryptotephra occurrence in the Amazon basin.

There has been much recent interest in tropical peatlands as they represent globally-important carbon sinks, support important ecosystems and are currently threatened by climate change and human activities^23^. It has been estimated that tropical peatlands contain approximately 88.6 Gt of carbon, equivalent to up to 19% of the global peatland carbon pool^23^,^24^ and can be found in both lowland and upland areas in SE Asia, Africa and Central and South America^25^,^26^,^27^. A variety of peatlands have recently been discovered in the subsiding Pastaza-Marañon basin in Western (Peruvian) Amazonia including minerotrophic palm swamps and ombrotrophic domed bogs^27^,^28^,^29^. The Pastaza-Marañon basin was recently identified as the most carbon-dense landscape in Amazonia, storing 892 ± 535 Mg C ha^−1^ 30. There have been a small number of studies of the ecology and paleoecology of Amazonia peatlands owing to their potential as archives of past environmental change^28^,^31^,^32^,^33^. Such studies are rare and thus important as they can provide a long-term baseline for recent climate changes in tropical Amazonia and globally. However, tropical peats are notoriously difficult to date due to the presence of large roots leading to deep biological alteration^32^.

Here we present a new discovery of a historic cryptotephra layer from a domed peatland in the Peruvian Amazon. The presence of this tephra has important implications for dating and correlating very recent peats and lake sediments in western Amazonia, and provides unambiguous evidence that Amazonia has been affected by volcanic ash fall in the very recent past.

Aucayacu (“water of the natives” or “water of the warriors”) is a domed peatland in western Peru that currently operates as an ombrotrophic ‘raised bog’ system^28^. It is situated on alluvial fan sediments between a stream of the Pastaza fan and the Tigre River (Fig. 1). The peatland began as a nutrient rich minerotrophic system that gradually became an ombrotrophic raised bog through its developmental history^28^. Aucayacu represents the deepest and oldest peatland that has been discovered in the Amazon basin (~7.5 m thick) and peat initiation at the site has been dated to c. 8870 cal. yr BP^28^. The vegetation of Aucayacu is characterised by ‘pole’ and ‘dwarf’ forest communities^33^.

## Methods

A peat core of length 1 m was extracted from the interior of Aucayacu peatland using a Russian D-section corer with a 50-cm-long chamber^34^,^35^. Peat moisture content and loss-on-ignition were calculated at 2 cm intervals following^36^ and peat humification was determined following^37^. The core was dated using AMS ^14^C dating of extracted wood and macrofossils. ^14^C dates were calibrated using IntCal13 38 in Clam v.2.2 39. Age-depth models using linear interpolation were constructed.

The core was analysed for tephra using the quick-burn technique^1^,^3^. After burning, the residue was sieved at 15 μm in an ultrasonic bath for 20 minutes to remove fine siliceous material, rinsed with deionised water, and the coarse fraction mounted onto slides. Tephra shard counts were conducted at 200× magnification on a standard Leica binocular microscope. Following detection of the peak tephra shard concentration, tephra was extracted for geochemical analysis following the density separation method of^40^. The peat sample was sieved between 80 and 15 μm. Further extraction was conducted using various densities of LST heavy liquid. A cleaning float of 2.0 g cm^−3^ was used to remove organic material a further float at of 2.2 g cm^−3^ was also required to remove abundant phytoliths. Finally tephra was floated off at 2.5 g cm^−3^ and rinsed thoroughly with deionised water. Samples were mounted onto glass slides using EpoThin resin, ground to expose the shards c.f.^41^ and polished to a 0.25 μm finish. Analysis was conducted by EPMA at the Tephra Analytical Unit, University of Edinburgh. Analysis setup followed the method of^12^, beam diameter was 5 μm with 15 kV and variable beam current 2 nÅ (Na, Mg, Al, Si, K, Ca, Fe) to 80 nÅ (P, Ti, Mn). Secondary glass standards, rhyolite (Lipari) and basalt (BCR-2G) were analysed before and after the unknown samples. The tephra geochemical data was compared with the Smithsonian’s Global Volcanism Program (2013) “Volcanoes of the World” database and the Large Magnitude Explosive Volcanic Eruptions (LaMEVE) database, which is part of VOGRIPA Project^42^. This resource and other published literature were searched for tephra geochemical data to identify a source volcano and/or eruption. Total Alkali-Silica (TAS) and geochemical bi-plots were constructed for comparison of the published tephra geochemical data with geochemical data from the AUC1 tephra.

## Results

**Figure 1:**
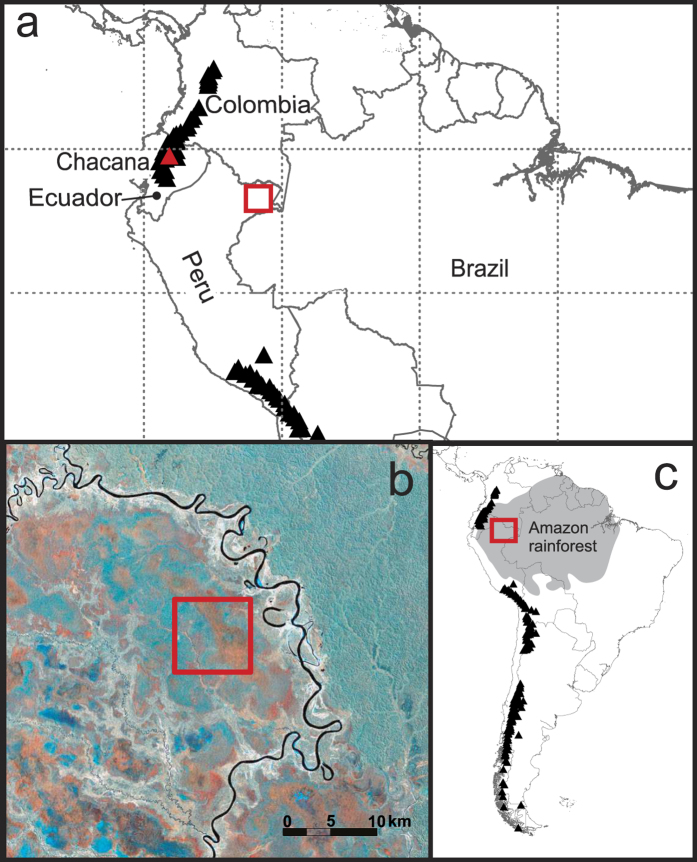
Maps showing the location of Aucayacu peatland, Loreto region, Peruvian Amazonia, (a) overview map of the approximate location of Aucayacu (red box) and the locations of volcanoes with known Holocene eruptions, the Chacana volcano, which is within the Eastern Cordillera is indicated in red, gridlines are at 10° intervals, (b) False colour Landsat TM RGB image (Orthorectified, WRS-2, Path 007, Row 063). Band 4 was assigned to red, band 5 was assigned to green and band 7 was assigned to blue. (**c**) Map indicating location of the field site in South America, again Holocene volcanoes are shown, shaded region indicates approximate forest cover. Maps were constructed using Arc Map 10.2.2. Landsat Data are free to download and available from the U.S. Geological Survey. Locations of Holocene locations downloaded from the Smithsonian Global Volcanism Program (http://www.volcano.si.edu/list_volcano_holocene.cfm#)

**Figure 2:**
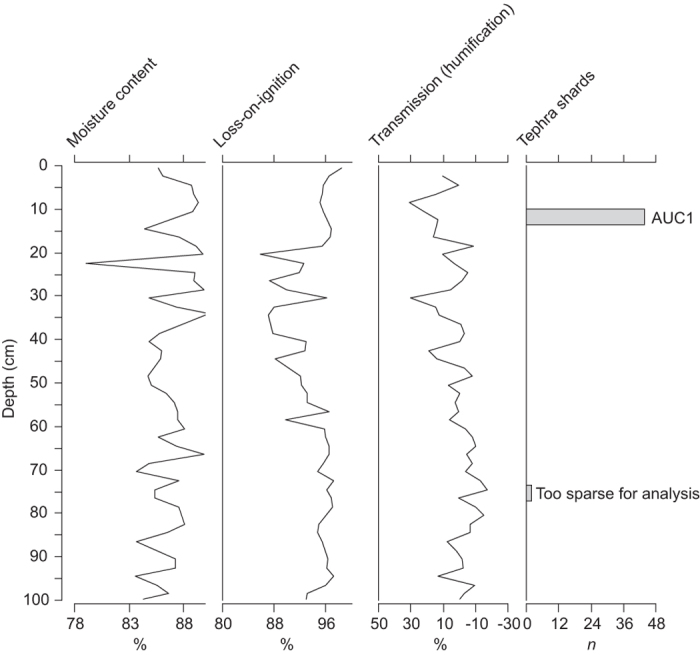
Core properties and tephrostratigraphy, *n *= number of tephra shards counted in the 5 cm^3^ sample, AUC1 is the tephra layer described in this study, a second tephra layer was detected but was not suitable for geochemical analysis due to a sparse number of tephra shards.

Figure 1 shows the location of Aucayacu peatland in Amazonia and volcanoes discussed in the text. In the 1-m core from Aucayacu there were no visible tephra layers; however, two microscopic tephra layers were encountered at 10–15 cm and 75–80 cm (Fig. 2). No tephra shards were identified in samples outside of these depths. The tephra layer at 10–15 cm (AUC1) had a sufficient concentration for analysis (44 shards 5 cm^−3^); however the lower layer only contained 2 shards and was not suitable for further analysis. The shards of AUC1 were all transparent and vesicular, with a mean size of 53 μm, median = 50 μm, maximum = 125 μm, and minimum 25 μm or less (n = 40). There is no clear event in the core properties (moisture content, loss-on-ignition or peat humification) that corresponds with the tephra layer. This (layer) is merely a trace of (volcanic) material and would not have been detected through visual means or analysis of basic core properties.

**Figure 3:**
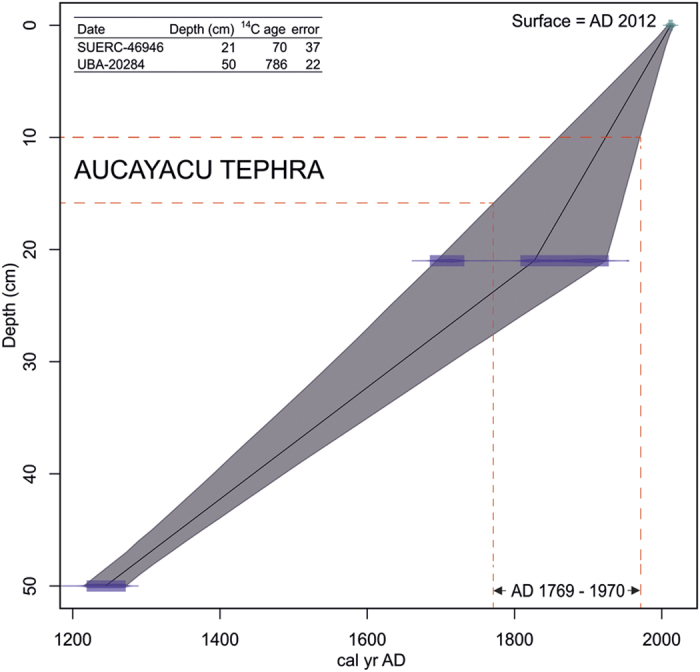
Age-depth model based on linear interpolation between the current surface and ^14^C dates at 21 and 50 cm. Based on our age depth model the peat depth containing the tephra is dated to between AD 1769 and 1970.

Age modelling, based on linear interpolation between the current surface (date of sampling = 2012) and two ^14^C dates, suggests a date range of AD 1769–1970 for the AUC1 tephra (Fig. 3). We note that the date at 21 cm runs to the modern period; however, the ^14^C date at 50 cm provides a solid constraint to the tephra being dated to within the last ~800 years.

## Discussion

Our discovery represents the first report of cryptotephra layers from Amazonia. Based on the distances travelled by other cryptotephras^13^,^14^ Aucayacu peatland is within cryptotephra fallout range for a moderate to large eruption from volcanoes in Peru, Ecuador and Colombia. The prevailing wind directions in the region of our study site are S/SE in the summer and N/NE in the winter^43^. However, there are no active Holocene volcanoes to the East of Aucayacu peatland. We therefore suggest that the tephra layers deposited at Aucayacu result from the eruptions of volcanoes along the Nazca and South American plate boundary which occurred during atypical (Westerly) wind conditions.

In an attempt to identify a source region and/or volcano for the AUC1 tephra we searched the Smithsonian Global Volcanism Database^44^ for volcanoes in Colombia, Ecuador or Peru, which had recorded eruptive activity around the time of the AUC1 tephra deposition. A total of 20 volcanoes have observed or dated eruptions during this time period (6 in Colombia, 9 in Ecuador and 5 in Peru). Geochemical analysis of the AUC1 tephra illustrates that it is rhyolitic with silica content >75%. Geochemical data is provided in supplementary table 1. Only one of these volcanoes, Chacana (Ecuador) is described as having a rhyolitic dominant rock type. However, there is evidence that volcanoes with a bulk rock geochemistry in the andesite range (as determined by XRF) can erupt rhyolitic glass which is the dominant constituent of distal cryptotephras^45^.

We examined the magnitude of eruptions around the time of the AUC1 tephra deposition. 16 of the volcanoes had no eruptions which were estimated to be larger than VEI 3, of the remaining volcanoes, 1 was in Ecuador (Cotopaxi), 1 in South Peru (Tutupaca erupted between AD 1787 and 1802^46^) and 1 in Colombia (Doña Juana erupted AD 1897–1906). There is evidence of distal ash deposition from Tutupaca at multiple locations including Arica (165 km from the vent)^46^ suggesting ash from this eruption was carried toward the South, the opposite direction to Aucayacu peatland. Although Doña Juana was active between 1897 and 1906 and activity peaked during 1899, contemporary reports do not indicate significant ash clouds^47^.

**Figure 4:**
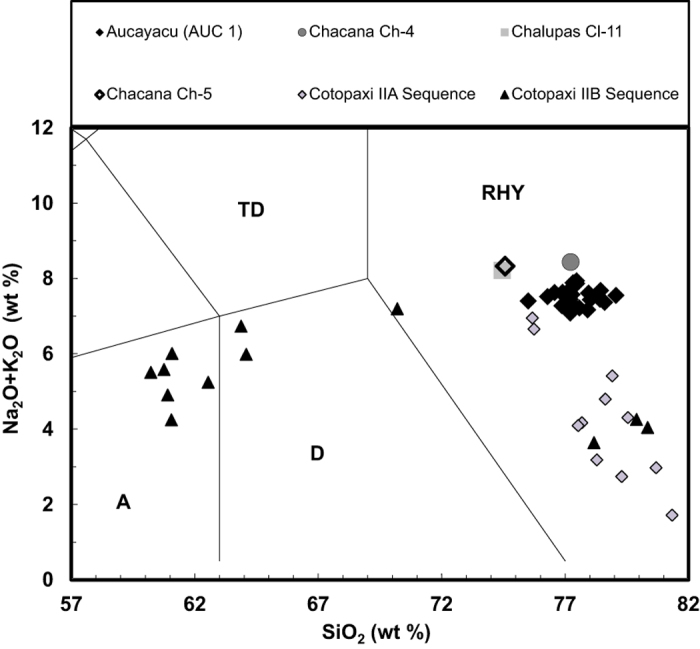
Total Alkali Silica (TAS) plot indicating the geochemistry of the Aucayacu tephra shards as determined by EPMA plotted against the whole rock geochemistry of volcanic rocks from the Chacana-Chalupas caldera region determined by XRF (X-ray fluorescence)^51^ and glass geochemical data for Cotopaxi determined by EPMA (Cotopaxi IIA and IIB sequences from the Holocene and late Pleistocene)^52^. Major element totals are normalised to 100%. Annotations follow standard terminology e.g. RHY = Rhyolite, D = Dacite, TD = Trachydacite^60^.

Following this initial search we focussed on the volcanoes of Ecuadorian Eastern Cordillera as: 1) They are closer to Aucayacu peatland than Colombian and Peruvian volcanoes (c. 5–600 km vs. 1500 km to Tutupaca and 700 km to Dona Juana); 2) Volcanoes in the Ecuadorian Eastern Cordillera have been highly active during the late Holocene, in particular Cotopaxi volcano which has three recorded eruptions with a magnitude of VEI 4 (AD 1744, 1768 and 1877^44^); 3) There is geochemical evidence to support the eruption of rhyolitic compositions from these volcanic systems in the past (Fig. 4).

Holocene Ecuadorian volcanism can be described by an East to West split with volcanoes in Eastern Cordillera generally more active than those in the West^48^. For this reason we focused our search to the East and specifically three large rhyolitic centres: Chalupas, Cotopaxi and Chacana (0–1°S, 78-79°W). Eruptions of Cotopaxi show characteristic rhyolitic and andesitic bimodal magmatism during the Holocene^48^ and multiple effusive and explosive eruptions of the volcano have been recorded in chronicles since 1534, with the largest historical event occurring in AD 1768 (VEI = 4)^49^. These eruptions were of andesitic bulk rock geochemistry. Less information is available about historical eruptive activity at the Chalupas volcano, which is adjacent Cotopaxi. The Chacana caldera complex is an eroded caldera complex of Pliocene-Holocene age^44^. Chacana stratovolcano (0.37° S, 78.25° W, elev. 4643 m) has been the source of multiple lava flows during the 18th century^50^.

**Figure 5:**
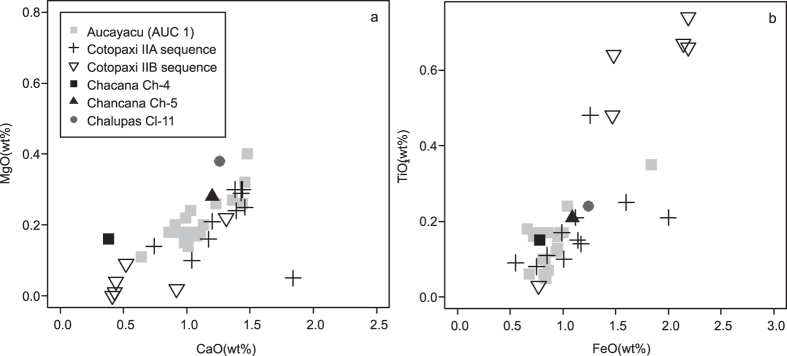
Co-variant plots of (a) CaO(%), MgO(%) (b) FeO(%), TiO_2_(%) values of the Aucayacu tephra glass shards as determined by EPMA plotted against the whole rock major values of volcanic rocks from the Chacana-Chalupas caldera region determined by X-ray fluorescence^51^ and glass geochemical data for Cotopaxi determined by EPMA (Cotopaxi IIA and IIB sequences from the Holocene and late Pleistocene)^52^. Major element totals are normalised to 100%.

Unfortunately only XRF bulk rock geochemical data is available for previous eruptions of Chalupas and Chacana^51^. Although XRF data indicates that these volcanoes have previously erupted bulk rock of rhyolite composition, due to the contamination of phenocrystals and microcrystals, the XRF data cannot be directly compared with the AUC 1 glass geochemistry (determined using EPMA), the data are plotted on Figs 4 and 5 for illustration only. There is an urgent need for the collection of representative proximal historical samples from Ecuador, Colombia and Peru which could be analysed via EPMA. There is some geochemical data based on EPMA of glass for Holocene and Late Pleistocene glasses from Cotopaxi^52^. Although this is similar to our AUC 1 data for some elements (Fig. 5), Cotopaxi rhyolites typically have a lower K_2_O value than the AUC 1 tephra (Fig. 4).

Our work shows that volcanic ash has been deposited more than once in Amazonia in the recent past. Given the close proximity of Amazonia to major volcanic chains of the Andes, the basin is likely to have been affected by volcanic ash fall throughout the Holocene. Analysis of the deeper peats (~7.5 m) at Aucayacu, for example, is likely to reveal a tephra record spanning a considerable proportion of the Holocene (peat initiation at c. 8870 cal. BP^28^). However, further work on a network of peatlands and lakes of Amazonia is needed to understand the long-term tephra record across the region. One problem is the current lack of a tephra geochemical database (e.g. Tephrabase for Europe^53^) for northern South America, making geochemical cross correlations difficult. Our work indicates that tephra glass shards are preserved for long periods of time and show no indication of either visible damage e.g. silica gel layer formation or pitting (cf.^40^) or geochemical changes (e.g. low total oxide values, fluctuation in alkaline elements) even in dynamic Amazonian peatlands with a pH of <4 and where the temperature (and thus rate of chemical and biological attack^54^) is likely to be higher than in northern peatlands.

Tephras may provide an important tool for correlating and dating palaeoenvironmental records from Amazonia and enable the determination of spatio-temporal variability in ecological dynamics and responses of ecosystems to changing climate. Furthermore, the AUC1 tephra may form an important isochron for dating and correlation of the recent part of tropical peatlands in western Amazonia which has implications for understanding recent changes, from the Little Ice Age to present. Tropical peatlands are highly dynamic in terms of biological activity (bioturbation) and hydrological regime. Amazonian peatlands are also affected by river flooding that is a significant factor for the reworking of microfossils. Tephra layers represent a discrete event in time; analysis of the structure of tephra layers in peat cores can offer a powerful tool to detect reworking with important implications for palaeoecological studies.

Tephra layers represent unequivocal evidence of deposition from ash clouds. As a result of the remote nature of much of Amazonia, written records of volcanic activity are unlikely to span more than a few centuries. In addition, proximal tephra records are often eroded or overlain by material from subsequent eruptions and therefore provide incomplete records of past volcanic activity. In these situations cryptotephras offer a complimentary approach to understanding the frequency of past explosive volcanic eruptions and the spatial extents of ash clouds^9^,^55^. Further research into cryptotephra deposits in the Amazon basin may provide some information on volcanic activity in this region.

Ash fall from volcanic eruptions is known to have significant impacts on vegetation that vary from short- to long-term^56^,^57^.The unequivocal evidence of ash clouds over Amazonia presented here highlights that the region has experienced the fallout products of volcanism. This raises the question of how much volcanic activity has impacted plant communities and plant function within this important ecosystem over time. Investigating the peat record could help to gain a further understanding of both the impact of volcanic activity on the plants of the Amazon basin and also of how wide-spread these impacts may be. When combined with palaeoecological records, cryptotephra layers offer the opportunity to consider plant community responses to volcanic events^58^,^59^.

As well as highlighting the opportunities for the development of tephrochronology for the dating of peatlands and lakes in Amazonia, this first discovery of cryptotephra in Amazonia indicates that volcanism has deposited volcanic ash and possibly volcanic gases over Amazonia. We suggest that this paper highlights the potential for future research into the tephrochronology and past ecology of this important region.

## Conclusions

1. We present information on the first microscopic tephra layer found in a peatland in western (Peruvian) Amazonia. Electron probe microanalysis provides geochemical data for the tephra that indicates a rhyolitic major element geochemistry. Radiocarbon dating suggests the AUC1 tephra fell between AD 1769 and 1970.
2. We suggest, based on the proximity to the Aucayacu peatland, geochemistry, and records of late Holocene volcanic activity that the most likely source for the AUC1 tephra is a volcano in the Ecuadorian Eastern Cordillera (0–1°S, 78-79°W).
3. This represents the first discovery of a historic microscopic tephra (cryptotephra) from Amazonia. The tephra layer may provide a new isochron for precise dating and correlation of palaeoenvironmental records from peatlands and lakes in western Amazonia.
4. The discovery of two tephra layers in the top 1 m of peat at Aucayacu demonstrates that cryptotephra layers can be preserved in the aggressive environments of Amazonian peatlands (low pH and high temperatures) and presents an opportunity for further research into the tephrochronology of this region.
5. Distal tephra layers in Amazonia may also provide much needed information on the frequency of volcanic activity and the characteristics of ash clouds in this region.
6. Further research is required; the presence of cryptotephra layers in Amazonian peatlands has important implications for understanding the influence of volcanic activity on the functioning of Amazonian vegetation communities. The possible impact of volcanic ash and gas fallout on the functioning of these communities is yet to be assessed.

## Additional Information

**How to cite this article**: Watson, E. J. *et al.* First discovery of Holocene cryptotephra in Amazonia. *Sci. Rep.* **5**, 15579; 10.1038/srep15579 (2015).

## Supplementary Material

Supplementary Dataset 1
