# Supplementary material for: First discovery of Holocene cryptotephra in Amazonia
Source: Sci Rep. 2015 Oct 23;5:15579. doi: 10.1038/srep15579 (PMC4616060; doi:10.1038/srep15579)
Supplement: Supplementary Dataset 1 [file srep15579-s1.doc]

Supplementary material for manuscript:

**First discovery of Holocene cryptotephra in Amazonia**

Elizabeth J. Watson*^1^

Graeme T. Swindles^1^

Ivan P. Savov^2^

Karen L. Bacon^1^

*^1^School of Geography, University of Leeds, Leeds, LS2 9JT, UK*

*^2^School of Earth and Environment, University of Leeds, Leeds, LS2 9JT, UK*

*Corresponding co-author

Supplementary Table 1: AUC1 Tephra geochemistry and standard data from EPMA

| Tephra | SiO_2_ | TiO_2_ | Al_2_O_3_ | FeO | MnO | MgO | CaO | Na_2_O | K_2_O | P_2_O_5_ | Total |
| --- | --- | --- | --- | --- | --- | --- | --- | --- | --- | --- | --- |
| AUC10 cm Population 1 | 76.33 | 0.12 | 12.79 | 0.93 | 0.03 | 0.27 | 1.34 | 4.23 | 2.77 | 0.04 | 98.86 |
| AUC10 cm Population 1 | 76.03 | 0.16 | 12.38 | 0.75 | 0.02 | 0.18 | 0.86 | 3.75 | 3.97 | 0.03 | 98.14 |
| AUC10 cm Population 1 | 75.29 | 0.16 | 11.59 | 0.74 | 0.01 | 0.17 | 0.89 | 3.85 | 3.30 | 0.02 | 96.02 |
| AUC10 cm Population 1 | 74.59 | 0.07 | 12.69 | 0.83 | 0.07 | 0.14 | 0.98 | 4.11 | 3.08 | 0.05 | 96.61 |
| AUC10 cm Population 1 | 74.73 | 0.10 | 12.27 | 0.78 | 0.02 | 0.25 | 1.18 | 4.24 | 2.71 | 0.04 | 96.32 |
| AUC10 cm Population 1 | 74.15 | 0.05 | 12.66 | 0.81 | 0.06 | 0.19 | 1.09 | 4.09 | 3.18 | 0.06 | 96.35 |
| AUC10 cm Population 1 | 75.71 | 0.15 | 11.56 | 0.73 | 0.03 | 0.17 | 0.83 | 3.56 | 3.53 | 0.03 | 96.32 |
| AUC10 cm Population 1 | 78.78 | 0.18 | 11.70 | 0.66 | 0.02 | 0.11 | 0.64 | 3.50 | 4.02 | 0.03 | 99.64 |
| AUC10 cm Population 1 | 77.00 | 0.10 | 12.49 | 0.82 | 0.03 | 0.24 | 1.02 | 4.27 | 2.81 | 0.04 | 98.83 |
| AUC10 cm Population 1 | 75.99 | 0.16 | 11.85 | 0.93 | 0.03 | 0.18 | 0.99 | 3.90 | 3.34 | 0.02 | 97.40 |
| AUC10 cm Population 1 | 73.41 | 0.06 | 12.98 | 0.79 | 0.07 | 0.16 | 1.04 | 4.13 | 3.18 | 0.05 | 95.87 |
| AUC10 cm Population 1 | 75.10 | 0.16 | 11.21 | 0.79 | 0.03 | 0.19 | 0.87 | 3.76 | 3.59 | 0.03 | 95.74 |
| AUC10 cm Population 1 | 73.73 | 0.06 | 12.52 | 0.65 | 0.06 | 0.16 | 0.94 | 4.10 | 3.12 | 0.05 | 95.40 |
| AUC10 cm Population 1 | 74.29 | 0.06 | 12.80 | 0.79 | 0.06 | 0.15 | 0.94 | 4.04 | 3.08 | 0.05 | 96.26 |
| AUC10 cm Population 1 | 77.14 | 0.17 | 12.32 | 1.00 | 0.03 | 0.22 | 0.99 | 3.87 | 3.99 | 0.04 | 99.76 |
| AUC10 cm Population 1 | 77.43 | 0.16 | 12.59 | 0.72 | 0.03 | 0.18 | 0.89 | 3.64 | 4.29 | 0.03 | 99.96 |
| AUC10 cm Population 1 | 74.79 | 0.35 | 12.77 | 1.82 | 0.06 | 0.40 | 1.47 | 4.16 | 3.17 | 0.06 | 99.05 |
| AUC10 cm Population 1 | 73.46 | 0.06 | 12.58 | 0.80 | 0.06 | 0.17 | 1.06 | 4.09 | 3.20 | 0.05 | 95.53 |
| AUC10 cm Population 1 | 75.31 | 0.24 | 12.97 | 1.03 | 0.02 | 0.26 | 1.42 | 4.09 | 3.33 | 0.04 | 98.71 |
| AUC10 cm Population 1 | 75.64 | 0.17 | 12.55 | 0.95 | 0.03 | 0.18 | 0.89 | 3.69 | 3.86 | 0.03 | 97.98 |
| AUC10 cm Population 1 | 76.56 | 0.17 | 12.00 | 0.84 | 0.02 | 0.15 | 0.96 | 3.66 | 3.81 | 0.04 | 98.21 |
| AUC10 cm Population 1 | 73.54 | 0.12 | 12.34 | 0.91 | 0.03 | 0.31 | 1.40 | 4.36 | 2.60 | 0.04 | 95.66 |
|  |  |  |  |  |  |  |  |  |  |  |  |
| Min | 73.41 | 0.05 | 11.21 | 0.65 | 0.01 | 0.11 | 0.64 | 3.50 | 2.60 | 0.02 | 95.40 |
| Max | 78.78 | 0.35 | 12.98 | 1.82 | 0.07 | 0.40 | 1.47 | 4.36 | 4.29 | 0.06 | 99.96 |
| Mean | 75.41 | 0.14 | 12.35 | 0.87 | 0.04 | 0.20 | 1.03 | 3.96 | 3.36 | 0.04 | 97.39 |
| Stdev | 1.42 | 0.07 | 0.49 | 0.24 | 0.02 | 0.07 | 0.21 | 0.25 | 0.47 | 0.01 | 1.55 |
|  |  |  |  |  |  |  |  |  |  |  |  |

| Standard | SiO_2_ | TiO_2_ | Al_2_O_3_ | FeO | MnO | MgO | CaO | Na_2_O | K_2_O | P_2_O_5_ | Total |
| --- | --- | --- | --- | --- | --- | --- | --- | --- | --- | --- | --- |
| BCR2g (before unknowns) | 54.72 | 2.25 | 13.29 | 12.26 | 0.20 | 3.66 | 7.15 | 3.11 | 1.76 | 0.36 | 98.76 |
| BCR2g (before unknowns) | 55.57 | 2.28 | 13.55 | 12.72 | 0.21 | 3.65 | 6.98 | 3.31 | 1.84 | 0.35 | 100.47 |
| BCR2g (before unknowns) | 54.63 | 2.27 | 13.71 | 12.43 | 0.20 | 3.81 | 7.19 | 3.23 | 1.83 | 0.35 | 99.65 |
| Lipari (before unknowns) | 74.60 | 0.07 | 13.27 | 1.58 | 0.06 | 0.06 | 0.75 | 4.24 | 5.22 | 0.01 | 99.86 |
| Lipari (before unknowns) | 73.69 | 0.08 | 12.80 | 1.51 | 0.07 | 0.03 | 0.74 | 4.08 | 5.13 | 0.00 | 98.14 |
| Lipari (before unknowns) | 75.67 | 0.08 | 12.86 | 1.50 | 0.08 | 0.05 | 0.74 | 4.45 | 5.27 | 0.00 | 100.70 |
|  |  |  |  |  |  |  |  |  |  |  |  |
| BCR2g (after unknowns) | 53.76 | 2.24 | 13.29 | 12.43 | 0.20 | 3.69 | 7.24 | 3.24 | 1.80 | 0.36 | 98.27 |
| BCR2g (after unknowns) | 53.77 | 2.23 | 13.07 | 12.33 | 0.19 | 3.75 | 7.28 | 3.38 | 1.78 | 0.36 | 98.15 |
| BCR2g (after unknowns) | 53.96 | 2.27 | 13.41 | 12.45 | 0.20 | 3.72 | 7.15 | 3.40 | 1.76 | 0.39 | 98.71 |
| BCR2g (after unknowns) | 53.52 | 2.23 | 13.56 | 12.27 | 0.19 | 3.73 | 7.25 | 3.31 | 1.75 | 0.36 | 98.16 |
| BCR2g (after unknowns) | 54.04 | 2.24 | 13.47 | 12.89 | 0.21 | 3.64 | 7.29 | 3.22 | 1.76 | 0.35 | 99.11 |
| BCR2g (after unknowns) | 54.15 | 2.24 | 13.08 | 12.39 | 0.20 | 3.70 | 7.31 | 3.31 | 1.87 | 0.35 | 98.60 |
| Lipari (after unknowns) | 74.90 | 0.09 | 12.79 | 1.67 | 0.06 | 0.03 | 0.75 | 4.32 | 5.25 | 0.00 | 99.87 |
| Lipari (after unknowns) | 74.42 | 0.07 | 13.01 | 1.51 | 0.06 | 0.05 | 0.77 | 4.20 | 5.23 | 0.01 | 99.34 |
| Lipari (after unknowns) | 73.94 | 0.08 | 12.83 | 1.41 | 0.06 | 0.06 | 0.74 | 4.11 | 5.27 | 0.01 | 98.50 |
| Lipari (after unknowns) | 74.14 | 0.07 | 12.91 | 1.59 | 0.06 | 0.05 | 0.74 | 4.25 | 5.17 | 0.00 | 98.99 |
| Lipari (after unknowns) | 74.44 | 0.07 | 12.85 | 1.55 | 0.07 | 0.05 | 0.77 | 4.35 | 5.11 | 0.01 | 99.27 |
| Lipari (after unknowns) | 74.82 | 0.07 | 13.02 | 1.71 | 0.07 | 0.05 | 0.69 | 4.22 | 4.99 | 0.00 | 99.64 |
